# Extended Nonnegative Tensor Factorisation Models for Musical Sound Source Separation

**DOI:** 10.1155/2008/872425

**Published:** 2008-05-29

**Authors:** Derry FitzGerald, Matt Cranitch, Eugene Coyle

**Affiliations:** ^1^Department of Electronic Engineering, Cork Institute of Technology, Cork, Ireland; ^2^School of Electrical Engineering Systems, Dublin Institute of Technology, Kevin Street, Dublin, Ireland

## Abstract

Recently, shift-invariant tensor factorisation algorithms have been proposed for the purposes of sound source separation of pitched musical instruments. However, in practice, existing algorithms require the use of log-frequency spectrograms to allow shift invariance in frequency which causes problems when attempting to resynthesise the separated sources. Further, it is difficult to impose harmonicity constraints on the recovered basis functions. This paper proposes a new additive synthesis-based approach which allows the use of linear-frequency spectrograms as well as imposing strict harmonic constraints, resulting in an improved model. Further, these additional constraints allow the addition of a source filter model to the factorisation framework, and an extended model which is capable of separating mixtures of pitched and percussive instruments simultaneously.

## 1. Introduction

The use of factorisation-based approaches for the separation of musical sound sources dates back to the early 1980s when Stautner used principal component analysis (PCA) to separate different tabla strokes [1]. However, it was not until the development of independent component analysis (ICA) [2] and techniques such as sparse coding [3, 4] and nonnegative matrix factorisation (NMF) [5, 6] that factorisation-based approaches received much attention for the analysis and separation of musical audio signals [7–11].

Factorisation-based approaches were initially applied to single channel separation of musical sources [7–10], where time-frequency analysis was performed on the input signal, yielding a spectrogram **X** of size *n* × *m*. This spectrogram was then factorised to yield a reduced rank approximation

(1) X≈X^=AS,

where **A** is of size *n* × *r* and **S** is of size *r* × *m*, with *r* less than *n* and *m*. In this case, the columns of **A** contain frequency basis functions, while the corresponding rows of **S** contain amplitude basis functions which describe when the frequency basis functions are active. Typically this is done on a magnitude or power spectrogram, and this approach makes the assumption that the spectrograms generated by the basis function pairs sum together to generate the mixture spectrogram. This does not take into account the effects of phase when the spectrograms are added together, and in the case of magnitude spectrograms this assumption is only true if the sources do not overlap in time and frequency, while it holds true on average for power spectrograms. Where the various techniques differ is in how this factorisation is achieved. Casey and Westner [7] used PCA to achieve dimensional reduction and then performed ICA on the retained principal components to achieve independent basis functions, while more recent work has focused on the use of nonnegativity constraints in conjunction with a suitable cost function [8, 9].

A commonly used cost function is the generalised Kullback-Leibler divergence proposed by Lee and Seung [5]:

(2) D(X∥X^)=∑ij(Xijlog⁡XijX^ij−Xij+X^ij)

which is equivalent to assuming a Poisson noise model for the data [12]. This cost function has been widely used due to its ease of implementation, lack of parameters, and the fact that it has been found to give reasonable results in many cases [13, 14]. A sparseness constraint can also be added to this cost function, and multiplicative update equations which ensure nonnegativity can be derived for these cost functions [15]. Other cost functions have been developed for factorisation of audio spectrograms such as that of Abdallah and Plumbley which assumes multiplicative gamma-distributed noise in power spectrograms [16]. A similar cost function recently proposed by Parry and Issa attempts to incorporate phase into the factorisation by using a probabilistic phase model [17, 18]. Families of parameterised cost functions have been proposed, such as the Beta divergence [19], and Csiszar's divergences [20]. The use of the Beta divergence for the separation of speech signals has been investigated by O'Grady [21], who also proposed a perceptually-based noise to mask ratio as a cost function.

Regardless of the cost function used, the resultant decomposition is linear, and as a result each basis function pair typically corresponds to a single note or chord played by a given pitched instrument. Therefore, in order to achieve sound source separation, some method is required to group the basis functions by source or instrument. Different grouping methods have been proposed in [7, 8], but in practice it is difficult to obtain the correct clustering for reasons discussed in [22].

### 1.1. Tensor Notation

When dealing with tensor notation, we use the conventions described by Bader and Kolda in [23]. Tensors are denoted using calligraphic uppercase letters, such as *𝒜*. Rather than using subscripts to indicate indexing of elements within a tensor or matrix, such as *𝒳*_*i*,*j*_, indexing of elements is instead notated by *𝒳*(*i*,*j*). When dealing with contracted product multiplication of two tensors, if *𝒲* is a tensor of size *I*_1_ × … × *I*_*N*_ × *J*_1_ × … × *J*_*M*_ and *𝒴* is a tensor of size *I*_1_ × … × *I*_*N*_ × *K*_1_ × … × *K*_*P*_, then contracted product multiplication of the two tensors along the first *N* modes is given by

(3) $$\begin{array}{ll} & {\langle 𝒲𝒴\rangle }_{\left\{1:N,1:N\right\}}(j_{1},\ldots ,j_{m},k_{1},\ldots ,k_{p})\\ & \;\;=\overset{I_{1}}{\underset{i_{1}=1}{\sum }}\cdots \overset{I_{N}}{\underset{i_{N}=1}{\sum }}𝒲(i_{1},\ldots ,i_{N},j_{1},\ldots ,j_{M})\\ & \;\;\;\;\;\;\;\;\times 𝒴(i_{1},\ldots ,i_{N},k_{1},\ldots ,k_{P}), \end{array}$$

where the modes to be multiplied are specified in the subscripts that are contained in the angle brackets.

Elementwise multiplication and division are represented by ⊗ and ⊘, respectively, and outer product multiplication is denoted by ∘. Further, for simplicity of notation, unless otherwise stated, we use the convention that :*k* denotes the tensor slice associated with the *k*th source, with the singleton dimension included in the size of the slice.

### 1.2. Tensor Factorisation

Recently, the above matrix factorisation techniques have been extended to tensor factorisation models to deal with stereo or multichannel signals by FitzGerald etal. [24] and Parry and Essa [25]. The signal model can be expressed as

(4) 𝒳≈𝒳^=∑b=1B𝒢:b∘𝒜:b∘𝒮:b,

where *𝒳* is an *r* × *n* × *m* tensor containing the spectrograms of the *r* channels, *𝒢* is an *r* × *B* matrix containing the gains of the *B* basis functions in each channel, *𝒜* is a matrix of size *n* × *B* containing a set of frequency basis functions, and *𝒮* is a matrix of size *m* × *B* containing the amplitude basis functions. In this case, :*b* is used to denote the *b*th column of a given matrix.

As a first approximation, many commercial stereo recordings can be considered to have been created by obtaining single-channel recordings of each instrument individually and then summing and distributing these recordings across the two channels, with the result that for any given instrument, the only difference between the two channels lies in the gain of the instrument [26]. The tensor factorisation model provides a good approximation to this case. The extension to tensor factorisation also provides another source of information which can be leveraged to cluster the basis functions, namely that basis functions belonging to the same source should have similar gains. However, as the number of basis functions increases it becomes more difficult to obtain good clustering using this information, as basis functions become shared between sources.

## 2. Shift-Invariant Factorisation Algorithms

The concept of incorporating shift invariance in factorisation algorithms for sound source separation was introduced in the convolutive factorisation algorithms proposed by Smaragdis [27] and Virtanen [28]. This was done in order to address a particular shortcoming of the standard factorisation techniques, namely that a single frequency basis function is unable to successfully capture sounds where the frequency content evolves with time, such as spoken utterances and drum sounds. To overcome this limitation, the amplitude basis functions were allowed to shift in time, with each shift capturing a different frequency basis function. When these frequency basis functions were combined, the result was a spectrogram of a given source that captured the temporal evolution of the frequency characteristics of the sound source.

Shift invariance in the frequency basis functions was later developed as a means of overcoming the problem of grouping the frequency basis functions to sources, particularly in the case where different notes played by the same instrument occurred over the course of a spectrogram [14, 29]. This shortcoming had been addressed by Vincent and Rodet using a nonlinear ISA approach [30], but this technique required pretraining of source priors before separation.

When incorporating shift invariance in the frequency basis functions, it is assumed that all notes played by a single pitched instrument consist of translated versions of a single frequency basis function. This single instrument basis function is then assumed to represent the typical frequency characteristics of that instrument. This is a simplification of the real situation, where in practice, the timbre of a given instrument does change with pitch [31]. Despite this, the assumption does represent a valid approximation over a limited pitch range, and this assumption has been used in many commercial music samplers and synthesisers, where a prerecorded note of a given pitch is used to generate other notes close in pitch to the original note. The principal advantage of using shift invariance in the frequency basis functions is that instead of having basis functions which must be grouped to their respective sources before separation can occur, as in standard NMF, the frequency shift invariant model allows individual instruments or sources to be modelled explicitly with each source having an individual slice of the tensors to be estimated.

Up till now, the incorporation of shift invariance in the frequency basis functions required the use of a spectrogram with log-frequency resolution, such as the constant *Q* transform (CQT) [32]. Alternatively, a log-frequency transform can be approximated by weighted summation of linear-frequency spectrogram bins, such as obtained from a short-time Fourier transform. This can be expressed as

(5) X=RY,

where **Y** is a linear-frequency spectrogram with *f* frequency bins and *t* time frames. **R** is a frequency weighting matrix of size *c* *f* × *f* which maps the *f* linear-frequency bins to *c* *f* log-frequency bins, with *c* *f* < *f* and **X** is a log-frequency spectrogram of size *c* *f* × *t*. It can be seen that **R** is a rectangular matrix and so no true inverse exists, making any mapping back from log-frequency resolution to linear frequency resolution only an approximate mapping.

If the frequency resolution of the log-frequency transform is set so that the center frequencies of the bands are given by *f*_*x*_ = *f*_0_ *β* ^*x*−1^, where *f*_*x*_ denotes the center frequency of the *x*th band, *β* = 2^1/12^, and *f*_0_ is a reference frequency, then the spacing of the bands will match that of the equal-tempered scale used in western music. A shift up or down by one bin will then correspond to a pitch change of one semitone.

In the context of this paper, translation of basis functions is carried out by means of translation tensors, though other formulations, such as the shift operator method proposed by Smaragdis [27] can be used. To shift an *n* × 1 vector, an *n* × *n* translation matrix is required. This can be generated by permuting the columns of the identity matrix. For example, in the case of shifting a basis function up by one, the translation matrix can be obtained from **I**(:,[*n*,1 : *n* − 1]), where the identity matrix is denoted by **I** and the ordering of the columns is contained in the square brackets where [*n*,1 : *n* − 1] indicates that *n* is the first element in the permutation, followed by entries of 1 : *n* − 1. For *Z* allowable translations, these translation matrices are then grouped into a translation tensor of size *n* × *Z* × *n*.

Research has also been done on allowing more general forms of invariance, such as that of Eggert etal. on transformation invariant NMF [33], where all forms of transformation such as translation and rotation are dealt with by means of a transformation matrix. However, their model has only been demonstrated on translation or shift invariance. Further, while a transformation matrix could potentially be used to allow the use of linear frequency resolution through the use of a matrix that stretches the spectrum, it has been noted elsewhere that this stretching is difficult to perform using a discrete linear frequency representation [13].

### 2.1. Shifted 2D Nonnegative Tensor Factorisation

All of the algorithms incorporating shift invariance can be seen as special cases of a more general model, shifted 2D nonnegative tensor factorisation (SNTF), proposed by FitzGerald [34], and separately by [35]. The SNTF model can then be described as

(6) $$𝒳\approx \overset{K}{\underset{k=1}{\sum }}{\langle {𝒢}_{:k}{\langle {\langle 𝒯{𝒜}_{:k}\rangle }_{\left\{3,1\right\}}{\langle {𝒮}_{:k}𝒫\rangle }_{\left\{3,1\right\}}\rangle }_{\left\{2:4,1:3\right\}}\rangle }_{\left\{2,2\right\}},$$

where *𝒳* is a tensor of size *r* × *n* × *m*, containing the magnitude spectrograms of each channel of the signal. *𝒢* is a tensor of size *r* × *K*, containing the gains of each of the *K* sources in each of the *r* channels. *𝒯* is an *n* × *z* × *n* translation tensor, which translates the instrument basis functions in *𝒜* up or down in frequency, where *z* is the number of translations in frequency, thereby approximating different notes played by a given source. *𝒜* is a tensor of size *n* × *K* × *p*, where *p* is the number of translations across time. *𝒮* is a tensor of size *z* × *K* × *m* containing the activations of the translations of *𝒜* which indicate when a given note played by a given instrument occurs, thereby generating a transcription of the signal. *𝒫* is an *m* × *p* × *m* translation tensor which translates the time activation functions contained in *𝒮* across time, thereby allowing time-varying source or instrument spectra. These tensors, their dimensions, and functions are summarised in Table 1 for ease of reference, as are all tensors used in subsequent models. If the number of channels is set to *r* = 1, and the allowable frequency translations *z* are also set to one, then the model collapses to that proposed by Virtanen in [28]. Similarly, setting *p* = 1 results in the model proposed in [36], while setting both *r* and *p* to one results in the model described in (4). In [34], the generalised Kullback-Leibler divergence is used as a cost function, and multiplicative update equations derived for *𝒢*, *𝒜*, and *𝒮*.

When using SNTF, a given pitched instrument is modelled by an instrument spectrogram which is translated up and down in frequency to give different notes played by the instrument. The gain parameters are then used to position the instrument in the correct position in the stereo field. A spectrogram of the *k*th separated source can then be estimated from (6) using only the tensor slices associated with the *k*th source. This spectrogram can then be inverted to a time-domain waveform by reusing the phase information of the original mixture signal, or by generating a set of phase information using the technique proposed by Slaney [37]. Alternatively, the recovered spectrogram can be used to generate a Wiener-type filter which can be applied to the original complex short-time Fourier transform.

As noted previously, the mapping from log-frequency to linear-frequency domains is an approximate mapping and this can have an adverse effect on the sound quality of the resynthesis. Various methods for performing this mapping and obtaining an inverse CQT have been investigated [38, 39]. However, a simpler method of overcoming this problem is to incorporate the mapping into the model. This can be done by replacing *𝒯* in (6) with 〈*ℛ* *𝒯*〉_{2,1}_, where *ℛ* is an approximate map from log to linear domains. This mapping can simply be the transpose of **R**, the mapping used in (5). Shift invariance is still implemented in the log-frequency domain, but the cost function is now measured in the linear-frequency domain. This is similar to the method proposed by O'Grady when using noise-to-mask ratio as a cost function [21]. O'Grady included the mapping from linear to Bark domain in his algorithm, as the cost function needed to be measured in the Bark scale domain. It was noted that this resulted in energy spreading in the magnitude spectrogram domain. In the modified SNTF algorithm, the opposite case applies, we wish to measure the cost function in the linear magnitude spectrogram domain, as opposed to a log-frequency domain, and the incorporation of the mapping results in less energy spreading in the frequency basis functions in the constant *Q* domain. It also has the advantage of performing the optimisation in the domain from which the final inversion to the time domain will take place. Despite this, the use of an approximate mapping still has adverse effects on the resynthesis quality.

In order to overcome these resynthesis problems, Schmidt etal. proposed using the spectrograms recovered to create masks which are then used to refilter the original spectrogram [40]. Schmidt etal. used a binary masking approach where bins were allocated to the source which had the highest power at that bin. In this paper, we use a refiltering method where the recovered source spectrogram is multiplied by the original mixture spectrogram as it was found that this gave better results than the previously described method.

## 3. Sinusoidal Shifted 2D Nonnegative Tensor Factorisation

While SNTF has been shown to be capable of separating mixtures of harmonic pitched instruments [34], a potential problem with the method is that there is no guarantee that the basis functions will be harmonic. A form of harmonic constraint, whereby the basis functions are only allowed to have nonzero values at regions which correspond to a perfectly harmonic sound, has been proposed by Virtanen [13] and later by Raczynski etal. [11], who used it for the purposes of multipitch estimation. However, with this technique, there is no guarantee that values returned in the harmonic regions of the basis functions will correspond to the actual shape that a sinusoid would have if present. It has also been noted by Raczynski that the structure returned when using this constraint may not always be purely harmonic as it is possible for the peaks to occur at points that are not at the centre of the harmonic regions.

An alternative approach to the problem of imposing harmonicity constraints on the basis functions is to note that the magnitude spectrum of a windowed sinusoid can be calculated directly in closed-form as a shifted and scaled version of the window's frequency response [41]. For example, using a Hann window, the magnitude spectrum of a sinusoid of frequency *f*_0_ = *h*2*π*/*f*_*s*_, where *h* is frequency in Hz, *f*_*s*_ is the sampling frequency in Hz, and *N* is the desired FFT, is given by

(7) $$X\left(x\right)=\left|0.5D\left(g\right)+0.25\{D_{1}\left(g\right)+D_{2}\left(g\right)\}\right|,$$

where *g* = *f*_*x*_ − *f*_0_, with *f*_*x*_ = *x*2*π*/*N* being the centre frequency of the *x*th FFT bin and where *D* is defined as

(8) $$D\left(g\right)=\frac{\sin\left(gN/2\right)}{\sin\left(g/2\right)},$$

with *D*_1_(*g*) = *D*(*g*−2*π*/*N*) and *D*_2_(*g*) = *D*(*g*+2*π*/*N*). It is then proposed to use an additive synthesis type model, where each note is modelled as a sum of sinusoids at integer multiples of the fundamental frequency of the note, with the relative strengths of the sinusoids giving the timbre of the note played. This spectral domain approach has been used previously to perform additive synthesis, in particular the inverse FFT method of Freed etal. [42].

For a given pitch and a given number of harmonics, the magnitude spectra of the individual sinusoids can be stored in a matrix of size *n* × *h*, where *n* is the number of bins in the spectrum, and *h* is the number of harmonics. This can be repeated for each of the allowed *z* notes, resulting in a tensor of size *n* × *z* × *h*. In effect, this tensor is a signal dictionary consisting of the magnitude spectra of individual sinusoids related to the partials of each allowable note. Again taking a Hann window as an example, the tensor can then be defined as

(9) $$\begin{array}{ll} & ℋ\left(x,i,j\right)=|0.5D\left(g_{xij}\right)+0.25\{D_{1}\left(g_{xij}\right)+D_{2}\left(g_{xij}\right)\}|, \end{array}$$

where *g*_*xij*_ = *f*_*x*_ − *f*_*i*,*j*_ with *f*_*i*,*j*_ = *h*_0_ *β* ^*i*−1^ *j*2*π*/*f*_*s*_, *h*_0_ is the frequency in hertz of the lowest allowable note and *β* is as previously defined in Section 2. This assumes equal-tempered tuning, but other tuning systems can also be used.

It is also possible to take into account inharmonicity in the positioning of the partials through the use of inharmonicity factors. For example, in the case of instruments containing stretched strings, *f*_*i*,*j*_ can be calculated as

(10) $$f_{i,j}=h_{0}\;\beta^{i-1}j2\pi \frac{\sqrt{1+\left(j^{2}-1\right)\alpha }}{f_{s}},$$

where *α* is the inharmonicity factor for the instrument in question [43]. In practice, the magnitude spectra will be close to zero except in the regions around *f*_*i*,*j*_, and so it is usually sufficient to calculate the values of *𝒯*(*x*,*i*,*j*) for ten bins on either side of *f*_*i*,*j*_ and to leave the remaining bins at zero. Further, the frequencies of the lowest partial of the lowest note, and the highest partial of the highest note place limits on the region of the spectrogram which will be modelled, and so spectrogram frequency bins outside of these ranges can be discarded. If a small number of harmonics are required, this can considerably reduce the number of calculations required, thereby speeding up the algorithm.

*ℋ* contains sets of harmonic partials all of equal gain. In order to approximate the timbres of different musical instruments, these partials must be weighted in different proportions. These weights can be stored in a tensor of size *h* × *K* × *p*, where *K* is the number of instruments and *p* is the number of translations across time, thereby allowing the harmonic weights to vary with time. Labeling the weights tensor as *𝒲*, the model can be described as

(11) $$𝒳=\overset{K}{\underset{k=1}{\sum }}{\langle {𝒢}_{:k}{\langle {\langle ℋ{𝒲}_{:k}\rangle }_{\left\{3,1\right\}}{\langle {𝒮}_{:k}𝒫\rangle }_{\left\{3,1\right\}}\rangle }_{\left\{2:4,1:3\right\}}\rangle }_{\left\{2,2\right\}}.$$

Using the generalised Kullback-Leibler divergence as a cost function, multiplicative update equations can be derived as

(12) $$\begin{array}{ll} {𝒢}_{:k} & ={𝒢}_{:k}⊗\frac{{\langle {\langle {\langle {\langle 𝒟ℋ\rangle }_{\left\{2,1\right\}}{𝒲}_{:k}\rangle }_{\left\{4,1\right\}}{𝒮}_{:k}\rangle }_{\left\{3:4,1:2\right\}}𝒫\rangle }_{\left\{2:4,3:1\right\}}}{{\langle {\langle {\langle {\langle 𝒪ℋ\rangle }_{\left\{2,1\right\}}{𝒲}_{:k}\rangle }_{\left\{4,1\right\}}{𝒮}_{:k}\rangle }_{\left\{3:4,1:2\right\}}𝒫\rangle }_{\left\{2:4,3:1\right\}}},\\ {𝒲}_{:k} & ={𝒲}_{:k}⊗\frac{{\langle {\langle \left({𝒢}_{:k}\circ ℋ\right)𝒟\rangle }_{\left\{\left[1,3\right],1:2\right\}}{\langle {𝒮}_{:k}𝒫\rangle }_{\left\{3,1\right\}}\rangle }_{\left\{\left[1,2,4\right],\left[1,2,4\right]\right\}}}{{\langle {\langle \left({𝒢}_{:k}\circ ℋ\right)𝒪\rangle }_{\left\{\left[1,3\right],1:2\right\}}{\langle {𝒮}_{:k}𝒫\rangle }_{\left\{3,1\right\}}\rangle }_{\left\{\left[1,2,4\right],\left[1,2,4\right]\right\}}},\\ {𝒮}_{:k} & ={𝒮}_{:k}⊗\frac{{\langle {\langle {\langle \left({𝒢}_{:k}\circ ℋ\right){𝒜}_{:k}\rangle }_{\left\{\left[2,5\right],\left[2,1\right]\right\}}𝒟\rangle }_{\left\{1:2,1:2\right\}}𝒫\rangle }_{\left\{2:3,\left[2,1\right]\right\}}}{{\langle {\langle {\langle \left({𝒢}_{:k}\circ ℋ\right){𝒜}_{:k}\rangle }_{\left\{\left[2,5\right],\left[2,1\right]\right\}}𝒪\rangle }_{\left\{1:2,1:2\right\}}𝒫\rangle }_{\left\{2:3,\left[2,1\right]\right\}}}, \end{array}$$

where $𝒟=𝒳⊘\hat{𝒳}$ and *𝒪* is an all-ones tensor with the same dimensions as *𝒳*, and all divisions are taken as elementwise.

These update equations are similar to those of SNTF, just replacing *𝒯* and *𝒜*, with a sinusoidal signal dictionary *ℋ*, and a set of harmonic weights *𝒲*, respectively. It is proposed to call this new algorithm *sinusoidal shifted 2D nonnegative tensor factorisation* (SSNTF) as it explicitly models the signal as the summation of weighted harmonically related sinusoids, in effect incorporating an additive synthesis model into the tensor factorisation framework. SSNTF can still be considered as shift invariant in frequency, as the harmonic weights are invariant to where in the frequency spectrum the notes occur.

An advantage of SSNTF is that the separation problem is now completely formulated in the linear-frequency domain, thereby eliminating the need to use an approximate mapping from log to linear frequency domains at any point in the algorithm, which removes the potential for resynthesis artifacts due to the mapping. Resynthesis of the separated time-domain waveforms can be carried out in a similar manner to that of SNTF, or alternatively, one can take advantage of the use of the additive synthesis model to reconstruct the separated signal using additive synthesis.

The SSNTF algorithm was implemented in Matlab using the Tensor Toolbox available from [44], as were all subsequent algorithms described in this paper. The cost function was always observed to decrease with each iteration. However, when running SSNTF, it was found that the best results were obtained when the algorithm was given an estimate of what frequency region each source was present in. This was typically done by giving an estimate of the pitch of the lowest note of each source. For score-assisted separation, such as that proposed by [45], this information will be readily available. The incorporation of this information has the added benefit of fixing the ordering of the sources in most cases. In cases where there is no score available, estimates can be obtained by running SNTF first and determining the pitch information from the recovered basis functions before running SSNTF. At present, research is being undertaken on devising alternate ways of overcoming this problem.

**Figure 1:**
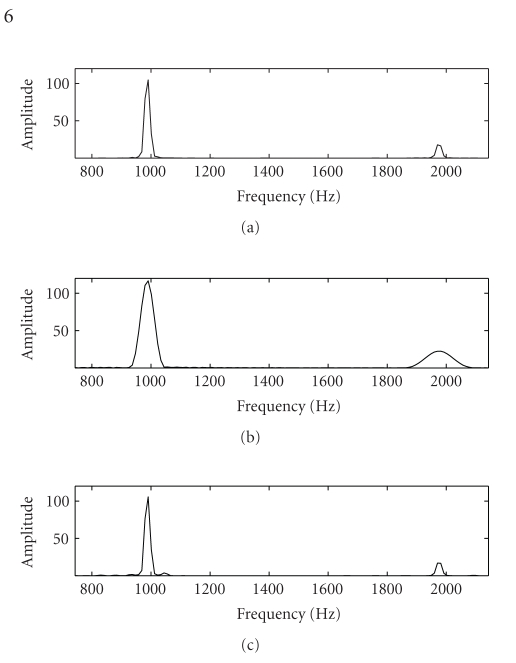
Spectra of flute note, original, SNTF, and SSNTF, respectively.

 As an example of the improved reconstruction that SSNTF can provide, Figure 1 shows the frequency spectrum of a flute note separated from a single channel mixture of flute and piano. SNTF and SSNTF were performed on this example using 9 translations in frequency and 5 translations in time. All other parameters were set as described later in Section 6. The first spectrum is that of the flute note taken from the original unmixed flute waveform, the second spectrum is that of the recovered flute note using SNTF, with the mapping from log to linear domains included in the model, while the third spectrum is that returned by SSNTF. It can be appreciated that the spectrum returned by SSNTF is considerably closer to the original than that returned by SNTF. This demonstrates the utility of using an approach which is formulated in the linear frequency domain.

**Figure 2:**
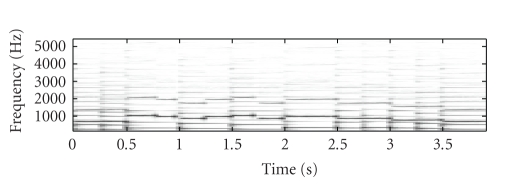
Spectrogram of piano and flute mixture.

**Figure 3:**
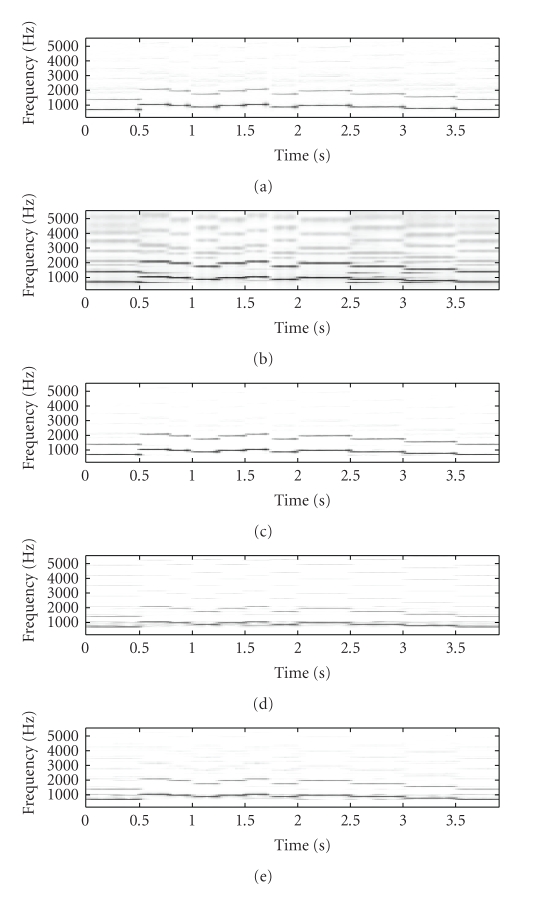
Spectrogram of flute signal, (a) original unmixed, (b) SNTF, (c) refiltered SNTF, (d) SSNTF, (e) source-filter SSNTF.

**Figure 4:**
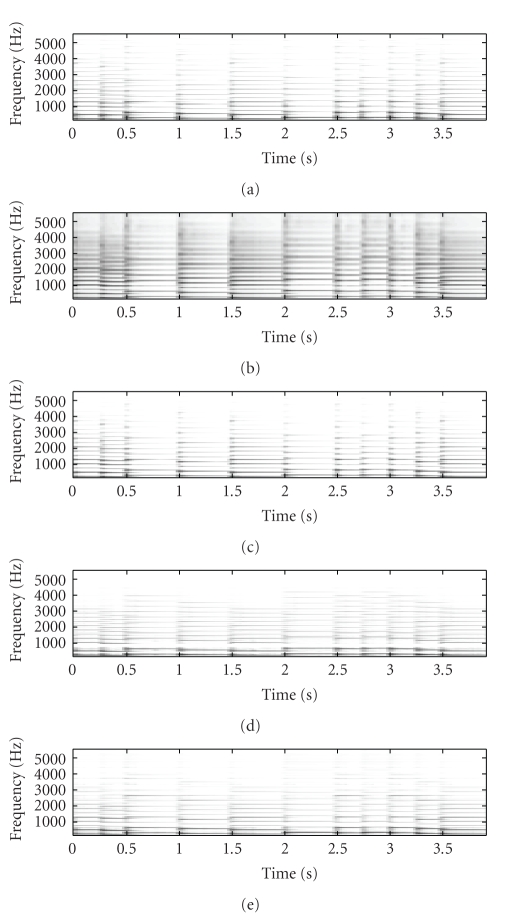
Spectrogram of piano signal, (a) original unmixed, (b) SNTF, (c) refiltered SNTF, (d) SSNTF, (e) source-filter SSNTF.

Figure 2 shows the original mixture spectrogram of piano and flute, while Figure 3(a) shows the unmixed flute spectrogram, with Figures 3(b), 3(c), and 3(d) showing the SNTF-separated flute spectrogram, the SNTF-separated flute spectrogram using refiltering, and the SSNTF-separated spectrogram, respectively. Figure 4(a) shows the unmixed piano spectrogram, with Figures 4(b), 4(c), and 4(d) showing the SNTF-separated piano spectrogram, the SNTF-separated piano spectrogram obtained using refiltering, and the SSNTF-separated spectrogram, respectively. It can be seen that the spectrograms recovered using SSNTF are considerably closer to the original spectrograms than that recovered directly from SNTF, where the smearing due to the approximate mapping from log to linear domains is clearly evident. Considerably improved recovery of the sources was also noted on playback of the separated SSNTF signals in comparison to those obtained using SNTF directly. The spectrograms obtained using SNTF in conjunction with refiltering can be also seen to be considerably closer to the original spectrograms than any of the other methods. However, on listening, the sound quality is still less than that obtained using SSNTF. Further, as will be seen later, the SNTF-based methods are not as robust as SSNTF-based methods.

It should also be noted that the addition of harmonic constraints imposes restrictions on the solutions that can be returned by the factorisation algorithms. This is of considerable benefit when incorporating additional parameters into the models, as will be seen in the following sections.

## 4. Source-Filter Modelling

As noted previously in Section 2, the use of a single shifted instrument basis function to model different notes played by an instrument is a simplification. In practice, the timbre of notes played by a given instrument changes with pitch, and this restricts the usefulness of shifted factorisation models. Recently, Virtanen and Klapuri proposed the incorporation of a source-filter model approach in the factorisation method as a means of overcoming this problem [46]. In the source-filter framework for sound production, the source is typically a vibrating object, such as a violin string, and the filter accounts for the resonant structure of the instrument, such as the violin body, which alters and filters the sound produced by the vibrating object. This approach had been used previously in both sound synthesis and speech coding [47, 48], but not in a factorisation framework.

When applied in the context of shifted instrument basis functions, the instrument basis function represents a harmonic excitation pattern which can be shifted up and down in frequency to generate different pitches. A single fixed filter is then applied to these translated excitation patterns, with the filter representing the instrument's resonant structure. This results in a system where the instrument timbre varies with pitch, resulting in a more realistic model. The instrument formant filters can be incorporated into the shifted tensor factorisation framework through a formant filter tensor *ℱ* of size *n* × *K* × *n*. In this case, the *k*th slice of *ℱ* is a diagonal matrix, with the instrument formant filter coefficients contained on the diagonal.

Unfortunately, attempts to incorporate the source-filter model into the SNTF framework were unsuccessful. The resultant algorithm had too many parameters to optimise and it was difficult to obtain good separation results. However, the additional constraints imposed by SSNTF were found to make the problem tractable. The resultant model can then be described as

(13) 𝒳≈𝒳^=∑k=1K〈𝒢:k〈〈ℛ:k𝒲:k〉{[2,4],[2,1]}𝒱:k〉{2:4,[2,1,3]}〉{2,2},

where *ℛ*_:*k*_ = 〈*ℱ*_:*k*_ *ℋ*〉_{3,1}_ and *𝒱*_:*k*_ = 〈*𝒮*_:*k*_ *𝒫*〉_{3,1}_.

Again using the generalised Kullback-Lieber divergence as a cost function, the following update equations were derived:

(14) $$\begin{array}{ll} {𝒢}_{:k} & ={𝒢}_{:k}⊗\frac{{\langle {\langle 𝒟{\langle {ℛ}_{:k}{𝒲}_{:k}\rangle }_{\left\{\left[2,4\right],\left[2,1\right]\right\}}\rangle }_{\left\{2,1\right\}}{𝒱}_{:k}\rangle }_{\left\{2:5,\left[4,2,1,3\right]\right\}}}{{\langle {\langle 𝒪{\langle {ℛ}_{:k}{𝒲}_{:k}\rangle }_{\left\{\left[2,4\right],\left[2,1\right]\right\}}\rangle }_{\left\{2,1\right\}}{𝒱}_{:k}\rangle }_{\left\{2:5,\left[4,2,1,3\right]\right\}}},\\ {ℱ}_{:k} & ={ℱ}_{:k}⊗\frac{{\langle {\langle {𝒢}_{:k}𝒟\rangle }_{\left\{1,1\right\}}{\langle {\langle ℋ{𝒲}_{:k}\rangle }_{\left\{3,1\right\}}{𝒱}_{:k}\rangle }_{\left\{2:4,1:3\right\}}\rangle }_{\left\{\left[1,3\right],2:3\right\}}}{{\langle {\langle {𝒢}_{:k}𝒪\rangle }_{\left\{1,1\right\}}{\langle {\langle ℋ{𝒲}_{:k}\rangle }_{\left\{3,1\right\}}{𝒱}_{:k}\rangle }_{\left\{2:4,1:3\right\}}\rangle }_{\left\{\left[1,3\right],2:3\right\}}},\\ {𝒲}_{:k} & ={𝒲}_{:k}⊗\frac{{\langle {\langle {\langle {𝒢}_{:k}{ℛ}_{:k}\rangle }_{\left\{2,2\right\}}𝒟\rangle }_{\left\{\left[1,3\right],1:2\right\}}{𝒱}_{:k}\rangle }_{\left\{\left[1,2,4\right],\left[2,1,4\right]\right\}}}{{\langle {\langle {\langle {𝒢}_{:k}{ℛ}_{:k}\rangle }_{\left\{2,2\right\}}𝒪\rangle }_{\left\{\left[1,3\right],1:2\right\}}{𝒱}_{:k}\rangle }_{\left\{\left[1,2,4\right],\left[2,1,4\right]\right\}}},\\ {𝒮}_{:k} & ={𝒮}_{:k}⊗\frac{{\langle {\langle {𝒢}_{:k}{\langle {ℛ}_{:k}{𝒲}_{:k}\rangle }_{\left\{\left[2,4\right],\left[2,1\right]\right\}}\rangle }_{\left\{2,2\right\}}{\langle 𝒟𝒫\rangle }_{\left\{3,1\right\}}\rangle }_{\left\{\left[1,3,5\right],1:3\right\}}}{{\langle {\langle {𝒢}_{:k}{\langle {ℛ}_{:k}{𝒲}_{:k}\rangle }_{\left\{\left[2,4\right],\left[2,1\right]\right\}}\rangle }_{\left\{2,2\right\}}{\langle 𝒪𝒫\rangle }_{\left\{3,1\right\}}\rangle }_{\left\{\left[1,3,5\right],1:3\right\}}}. \end{array}$$

**Figure 5:**
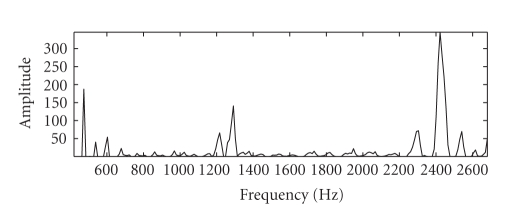
Filter returned for flute when using source-filter SSNTF.

Figure 5 shows the filter recovered for the flute from the example previously discussed in Section 3. It can be seen that the recovered filter consists of a series of peaks as opposed to a smooth formant-like filter. This is due to a combination of two factors, firstly, the small number of different notes played in the original signal, and secondly, the harmonic constraints imposed by SSNTF. This results in a situation where large portions of the spectrum will have little or no energy, and accordingly the filter models these regions as having little or no energy.

On listening to the resynthesis, there was a marked improvement in the sound quality of the flute in comparison with SSNTF, with less high-frequency energy present. The resynthesis of the piano also improved, though less so than that of the flute. Figures 3(e) and 4(e) show the spectrograms recovered using source-filter SSNTF for the flute and piano, respectively. It can be observed that the flute spectrogram is closer to the original than either SNTF or SSNTF, with no smearing and a reduced presence of higher harmonics in comparison to SSNTF, which is in line with what was observed on listening to the resynthesis. In comparison to the SNTF and refiltering approach, source-filter SSNTF has retained more high-frequency information than the refiltered approach, and can be seen to be closer to the original spectrogram. In the case of the piano, the refiltered spectrogram contains more high-frequency information than the source-filter SSNTF approach, which is closer to the original piano spectrogram. On listening, the source-filter SSNTF approach also outperforms the refiltered SNTF approach.

**Figure 6:**
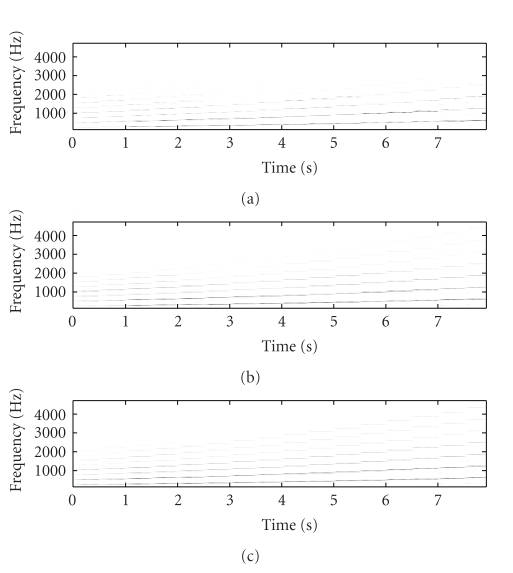
Spectrograms for (a) original flute spectrogram, (b) spectrogram recovered using source-filter SSNTF, and (c) spectrogram recovered using SSNTF.

**Figure 7:**
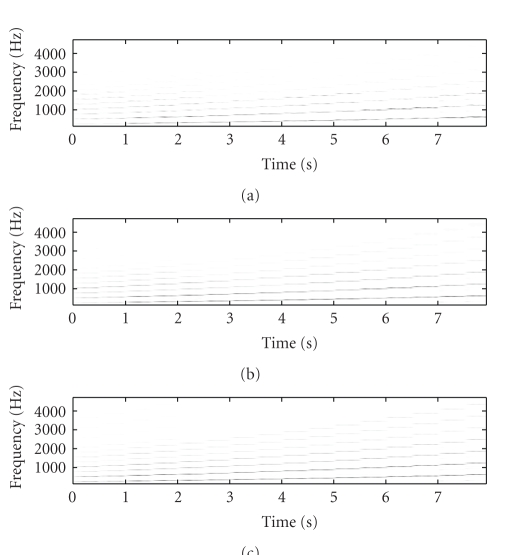
Filter returned for solo flute example in Figure 6 when using source-filter SSNTF.

As a further example of source-filter SSNTF, Figure 6(a) shows the spectrogram of a flute signal consisting of 16 notes, one semitone apart played in ascending order, while Figures 6(b) and 6(c) show the spectrogram recovered using source-filter SSNTF and SSNTF, respectively. It can be seen that the source-filter method has returned a spectrogram closer to the original, with less high-frequency information than SSNTF. Figure 7 shows the source-filter associated with Figure 6(b). It can be seen that in this case, where 16 successive notes are played, the source-filter is smoother, as would be expected for a formant-like filter, but as the harmonics get further apart, evidence of peakiness similar to that in Figure 5 becomes more evident.

The above examples demonstrate the utility of using the source-filter approach as a means of improving the accuracy of the SSNTF model. This is bourn out in the improved resynthesis of the separated sources. 

## 5. Separation of Pitched and Nonpitched Instruments

Musical signals, especially popular music, typically contain unpitched instruments such as drum sounds in addition to pitched instruments. While allowing shift invariance in both frequency and time is suitable for separating mixtures of pitched instruments, it is not suitable for dealing with percussion instruments such as the snare and kick drums, or other forms of noise in general. These percussion instruments can be successfully captured by algorithms which allow shift invariance in time only without the use of frequency shift invariance. In order to deal with musical signals containing both pitched and percussive instruments or contain additional noise, it is necessary to have an algorithm which handles both these cases. This can be done by simply adding the two models together. This has previously been done by Virtanen in the context of matrix factorisation algorithms [13], who also noted that the resulting model was too complex to obtain good results without the addition of additional constraints. In particular, the use of a harmonicity constraint was required, though in this case it was based on zeroing instrument basis functions in areas where no harmonic activity was expected, as opposed to the additive synthesis-based technique proposed in this paper.

Extending the concept to the case of tensor factorisation techniques results in a generalised tensor factorisation model for the separation of pitched and percussive instruments, which still allows the use of a source-filter model for pitched instruments. The model can be described by

(15) 𝒳≈𝒳^=∑k=1K〈𝒢:k〈〈ℛ:k𝒲:k〉{[2,4],[2,1]}𝒱:k〉{2:4,[2,1,3]}〉{2,2} +∑l=1L〈ℳ:l〈ℬ:k〈𝒞:l𝒬〉{2,1}〉{2:3,1:2}〉{2,2},

where *ℳ* is a tensor of size *r* × *L*, which contains the gains of each of the *L* percussive sources, *ℬ* is a tensor of size *n* × *L* × *q*, where *q* is the number of allowable time shifts for the percussive sources, *𝒞* is a tensor of size *L* × *m*, and *𝒬* is a translation tensor of size *m* × *q* × *m*. Multiplicative update equations, based on the generalised Kullback-Leibler divergence can then be derived for these additional parameters, while update equations for all other parameters are as given in Section 4. The additional update equations are given by

(16) $$\begin{array}{ll} {ℳ}_{:l} & ={ℳ}_{:l}⊗\frac{{\langle 𝒟{\langle ℬ{\langle 𝒞𝒬\rangle }_{\left\{2,1\right\}}\rangle }_{\left\{2:3,1:2\right\}}\rangle }_{\left\{2:3,\left[1,3\right]\right\}}}{{\langle 𝒪{\langle ℬ{\langle 𝒞𝒬\rangle }_{\left\{2,1\right\}}\rangle }_{\left\{2:3,1:2\right\}}\rangle }_{\left\{2:3,\left[1,3\right]\right\}}},\\ {ℬ}_{:l} & ={ℬ}_{:l}⊗\frac{{\langle {\langle {ℳ}_{:l}𝒟\rangle }_{\left\{1,1\right\}}{\langle 𝒞𝒬\rangle }_{\left\{2,1\right\}}\rangle }_{\left\{\left[1,3\right],\left[1,3\right]\right\}}}{{\langle {\langle {ℳ}_{:l}𝒪\rangle }_{\left\{1,1\right\}}{\langle 𝒞𝒬\rangle }_{\left\{2,1\right\}}\rangle }_{\left\{\left[1,3\right],\left[1,3\right]\right\}}},\\ {𝒞}_{:l} & ={𝒞}_{:l}⊗\frac{{\langle {\langle {ℳ}_{:l}ℬ\rangle }_{\left\{2,2\right\}}{\langle 𝒟𝒬\rangle }_{\left\{3,3\right\}}\rangle }_{\left\{\left[1,3,4\right],\left[1,2,4\right]\right\}}}{{\langle {\langle {ℳ}_{:l}ℬ\rangle }_{\left\{2,2\right\}}{\langle 𝒪𝒬\rangle }_{\left\{3,3\right\}}\rangle }_{\left\{\left[1,3,4\right],\left[1,2,4\right]\right\}}}. \end{array}$$

The individual sources can be separated as before, but the algorithm can also be used to separate the pitched instruments from the unpitched percussive instruments or vice-versa by resynthesising the relevant section of the model. It can also be used as a means of eliminating noise from mixtures of pitched instruments by acting as a type of “garbage collector,” which can improve resynthesis quality in some cases. It can also be viewed as being analogous to the additive plus residual sinusoidal analysis techniques described by Serra [49] in that it allows the pitched or sinusoidal part of the signal to be resynthesised separately from the noise part of the signal.

**Figure 8:**
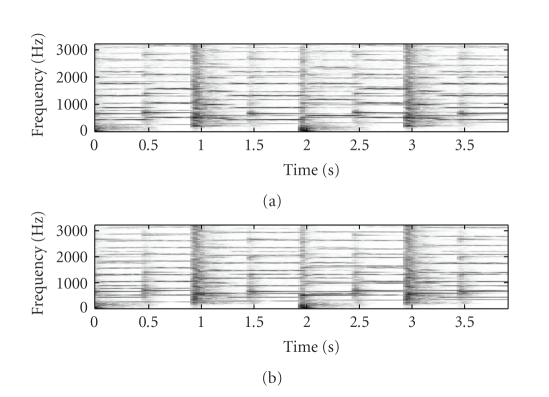
Mixture spectrograms of piano, flute, trumpet, snare, hi-hats, and kick drum.

**Figure 9:**
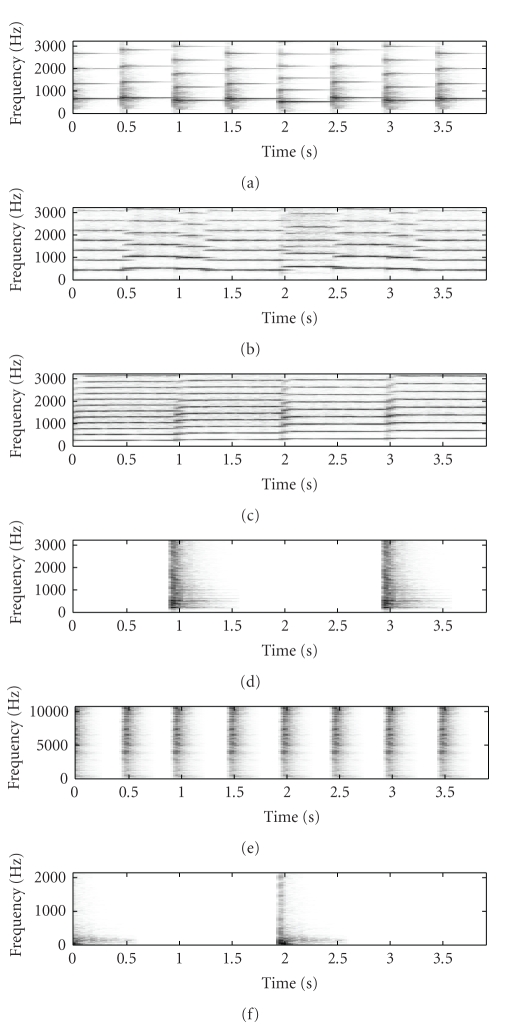
Original spectrograms of (a) piano, (b) flute, (c) trumpet, (f) snare, (g) hi-hats, and (h) kick drum.

**Figure 10:**
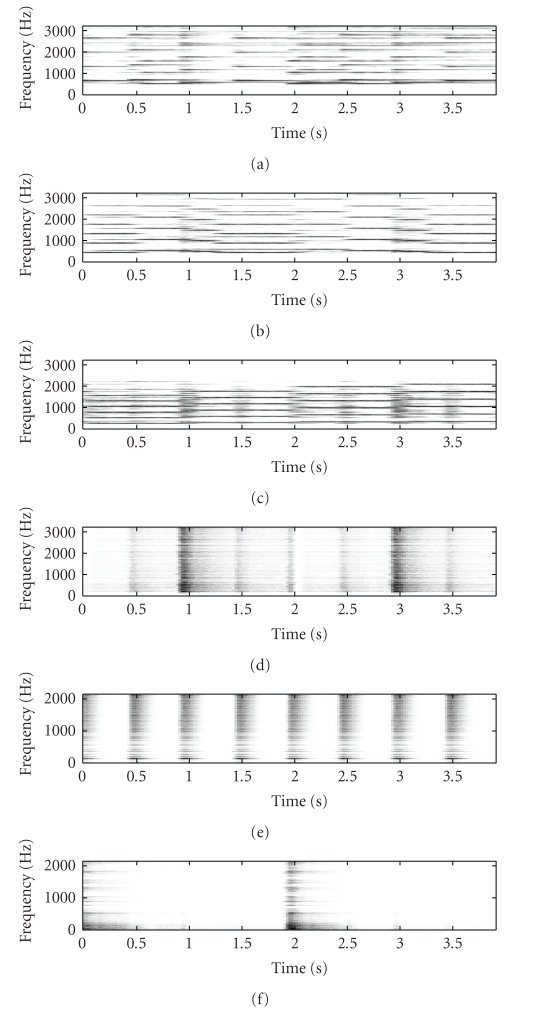
Separated spectrograms of (a) piano, (b) flute, (c) trumpet, (f) snare, (g) hi-hats and (h) kick drum.

As an example of the use of the combined model, Figure 8 shows the mixture spectrograms obtained from a stereo mixture containing three pitched instruments, piano, flute, and trumpet, and three percussion instruments, snare, hi-hats, and kick drum, while Figure 9 shows the original unmixed spectrograms for those sources, respectively. The piano, snare, and kick drum were all panned to the center, with the hi-hats and flute panned midleft and the trumpet midright. Figure 10 shows the separated spectrograms obtained using the combined model. It can be seen that the sources have been recovered well, with each individual instrument identifiable, though traces of other sources can be seen in the spectrograms. This is most evident where traces of the hi-hats are visible in the snare spectrogram, but the snare clearly predominates. On listening to the results, traces of the flute can also be heard in the piano signal, and the timbres of the instruments have been altered, but are still recognisable as being the instrument in question. The example also highlights another advantage of tensor factorisation models in general, namely the ability to separate instruments which have the same position in the stereo field. This is in contrast to algorithms such as Adress and DUET, which can only separate sources if they occupy different positions in the stereo field [26, 50].

## 6. Performance Evaluation

The performances of SNTF, SNTF using refiltering, SSNTF, source-filter SSNTF, and source-filter SSNTF with noise basis functions in the context of modelling mixtures of pitched instruments were compared using a set of 40 test mixtures. In the case of source-filter SSNTF with noise basis functions, two noise basis functions were learned in order to aid the elimination of noise and artifacts from the harmonic sources. The 40 test signals were of 4 seconds duration and contained mixtures of melodies played by different instruments and created by using a large library of orchestral samples [51]. Samples from a total of 15 different orchestral instruments were used. A wide range of pitches were covered, from 87 Hz to 1.5 kHz, and the melodies played by the individual instruments in each test signal were in harmony. This was done to ensure that the test signals contained extensive overlapping of harmonics, as this occurs in most real world musical signals. In many cases, the notes played by one instrument overlapped notes played by another instrument to test if the algorithms were capable of discriminating notes of the same pitch played by different instruments.

The 40 test signals consisted of 20 single channel mixtures of 2 instruments and 20 stereo mixtures of 3 instruments, and these mixtures were created by linear mixing of individual single channel instrument signals. In the case of the single channel mixtures, the source signals were mixed with unity gain, and in the case of the stereo mixtures, mixing was done according to

(17) $$\left(\begin{array}{c} x_{1}(t)\\ x_{2}(t) \end{array}\right)=\left(\begin{array}{ccc} 0.75 & 0.5 & 0.25\\ 0.25 & 0.5 & 0.75 \end{array}\right)\left(\begin{array}{c} s_{1}(t)\\ s_{2}(t)\\ s_{3}(t) \end{array}\right),$$

where *x*_1_(*t*) and *x*_2_(*t*) are the left and right channels of the stereo mixture and *s*_1_(*t*) represents the first single channel instrument signal and so on.

Spectrograms were obtained for the mixtures, using a short-time Fourier transform with a Hann window of 4096 samples, with a hopsize of 1024 samples between frames. All variables were initialised randomly, with the exception of the frequency basis functions for SNTF-based separation, which were initialised with harmonic basis functions at the frequency of the lowest note played by each instrument in each example. This was done to put SNTF on an equal footing with the SSNTF-based algorithms, where the pitch of the lowest note of each source was provided. The number of allowable notes was set to the largest pitch range covered by an instrument in the test signal and the number of harmonics used in SSNTF was set to 12. The algorithms were run for 300 iterations, and the separated source spectrograms were estimated by carrying out contracted tensor multiplication on the tensor slices associated with an individual source. The recovered source spectrograms were resynthesised using the phase information from the mixture spectrograms. The phase of the channel where the source was strongest was used in the case of the stereo mixtures.

Using the original source signals as a reference, the performance of the different algorithms were evaluated using commonly used metrics, namely the signal-to-distortion ratio (SDR), which provides an overall measure of the sound quality of the source separation, the signal-to-interference ratio (SIR), which measures the presence of other sources in the separated sounds, and the signal-to-artifacts ratio (SAR), which measures the artifacts present in the recovered signal due to separation and resynthesis. Details of these metrics can be found in [52] and a Matlab toolbox to calculate these measures is available from [53]. As noted previously in Section 3, the provision of the lowest pitch note for each source was sufficient to determine the correct source ordering for all the SSNTF-based algorithms. In the case of the SNTF-based algorithms, the ordering of the sources was determined by associating a separated source with the original source which resulted in the best SIR score. This matching procedure was then checked manually to ensure no errors had occurred.

**Figure 11:**
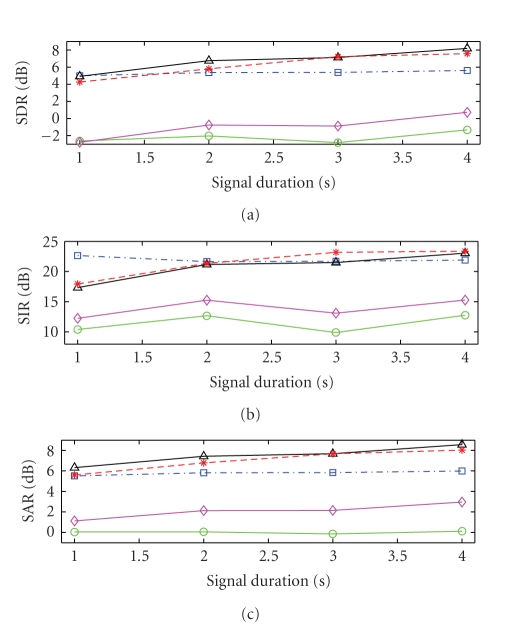
Performance evaluation of SNTF (circle solid), refiltered SNTF (diamond solid), SSNTF (square dash-dotted), source-filter SSNTF (triangle solid), and source-filter SSNTF (star dashed) with noise basis functions for various signal durations.

A number of different tests were run to determine the effect of signal duration on the performance of the algorithms and to determine the effect of using different numbers of allowable shifts in time. For the tests on signal duration, the mixture signals were truncated to lengths of 1, 2, 3, and 4 seconds in length, the number of time shifts was set to 5, and the performance of the algorithms was evaluated. A summary of the results obtained are shown in Figure 11. The results were obtained by averaging the metrics obtained for each separated source to give an overall score for each test mixture. The results for each mixture were then averaged to yield the data shown in the figure. It can be seen that the SSNTF-based algorithms all clearly outperform SNTF-based methods in all cases, though the use of refiltering does improve the performance of SNTF. It can also be seen that signal duration does not have much effect on the results obtained from SSNTF, with the results remaining relatively constant with signal duration, showing that SSNTF can capture harmonic sources even at relatively short signal durations.

In the case of the algorithms incorporating source filtering, performance improved with increased signal duration. This is particularly evident in the case of the SIR metric. This demonstrates that longer signal durations are required to properly capture filters for each instrument. This is to be expected as increased numbers of notes played by each instrument provide more information on which to learn the filter, while the harmonic model with fewer parameters does not require as much information for training. It should be noted that this trend was less evident in the stereo mixtures than in the mono mixtures, suggesting that the spatial positioning of sources in the stereo field may effect the ability to learn the source filters. This can possibly be tested by measuring the separation of the sources while varying the mixing coefficients and is an area for future investigation. Nonetheless, it can be seen that at longer durations the source-filter approaches outperform SSNTF, with the basic source-filter model performing better in terms of SDR and SAR, while the source-filter plus noise approach performs better in terms of SIR.

**Figure 12:**
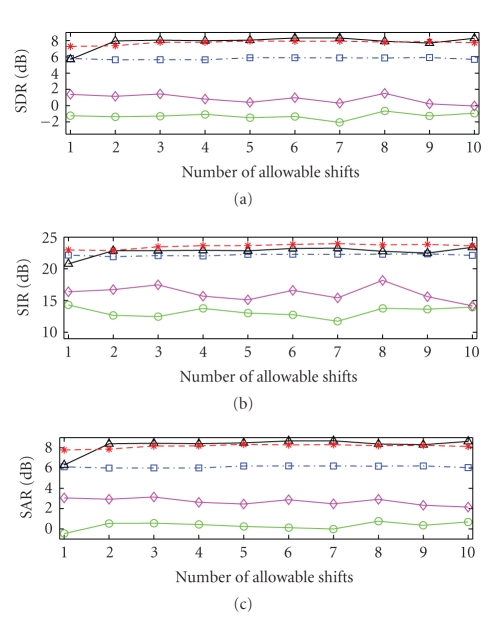
Performance Evaluation of SNTF (circle solid), refiltered SNTF (diamond solid), SSNTF (square dash-dotted), Source-Filter SSNTF (triangle solid) and Source-Filter SSNTF (star dashed) with noise basis functions for various allowable shifts in time.

The results from testing the effect of the number of time shifts on the separation of the sources are shown in Figure 12. These were obtained using the same procedure used for the previous tests. The number of allowable shifts ranged from 1 to 10, which corresponds to a maximum shift in time of approximately 0.2 second. Once again, the SSNTF-based algorithms clearly outperform SNTF-based approaches, regardless of the shift. However, it can be seen that for both SSNTF and the source-filter plus noise approach, performance is relatively constant with the number of allowable shifts, there is a small improvement in performance up until 7 shifts and beyond this performance degrades slightly. In the case of source-filter SSNTF, there is a noticeable improvement when going from one to two shifts, but beyond this there is little or no variation in performance with increased numbers of shifts. On investigating, this was found to be mainly evident in the stereo mixtures, with the performance of the mono mixtures remaining relatively constant, again highlighting the need to investigate the performance of the algorithms under different mixing coefficients. Overall, it can be seen that the performance of the algorithms is in line with that observed when varying signal duration, with the source-filter plus noise approach performing best in terms of SIR, while source-filter SSNTF performs better in terms of SDR and SAR. Further, the results suggest that in many cases, a single set of harmonic weights can be used to characterise pitched instruments without the need to incorporate timbral change with time.

On listening to the separated sources, the SSNTF-based approaches clearly outperform SNTF. It should be noted that in some cases, SNTF using refiltering resulted in audio quality comparable to the SSNTF-based approaches, however this was only in a small number of examples. In the majority of cases the addition of the source-filter improves on the results obtained by SSNTF. On comparing the source-filter approach to the source-filter plus noise model, it was observed that the results varied from mixture to mixture, with a considerable improvement in resynthesis quality of some sources and a reduction of quality in other cases, while in a large number of tests no major differences could be heard in the results. This shows that in many cases for clean mixture signals of pitched instruments, there is no need to incorporate noise basis functions. Nevertheless, the use of noise basis functions is still useful in the presence of noise or percussion instruments. It should also be noted that in half of the test mixtures SNTF did not manage to correctly separate the sources, which, in conjunction with the distortion due to the smearing of the frequency bins due to the mapping from log to linear frequency, goes a long way towards explaining the negative SDR and SIR scores. While SNTF using refiltering resulted in improved resynthesis in the cases where the sources had been separated correctly, it also suffered from the reliablity issues of the underlying SNTF technique and this is reflected in the poor scores for all metrics. This indicates that the SSNTF-based techniques are considerably more robust than SNTF-based techniques.

The separated sources can also be resynthesised via an additive synthesis approach, and on listening, the results obtained were comparable to those obtained from the spectrogram-based resynthesis. However, as the additive synthesis approach uses different phase information than the spectrogram-based resynthesis, the results are not comparable using the metrics used in this paper. This highlights the need to develop a set of perceptually-based metrics for sound source separation and is an area for future research.

Also investigated was the goodness of fit of the models to the original spectrogram data, as measured by the cost function. It was observed that the results obtained for SSNTF were on average 64% smaller than those for SNTF, despite the fact that SSNTF has a smaller number of free parameters, as the number of harmonics was considerably smaller than the number of frequency bins used in the constant *Q* spectrogram for SNTF. This highlights the benefits of using an approach solely formulated in the linear frequency domain. Using source-filter SSNTF, with an additional *K*×*n* parameters over SSNTF, resulted in an average reduction in the cost function of 76% in comparison to SNTF, and a reduction of 33% in comparison to SSNTF.

Overall it can be seen that the methods proposed in this paper offer a considerable improvement over previous separation methods using SNTF. Large improvements can be seen in the performance metrics over the previous SNTF method, and it can also be seen that the proposed models result in an improved fit to the original data.

## 7. Conclusions

The use of shift-invariant tensor factorisations for the purposes of musical sound source separation, with a particular emphasis on pitched instruments, has been discussed, and problems with existing algorithms were highlighted. The problem of grouping notes to sources can be overcome by incorporating shift invariance in frequency into the factorisation framework, but comes at the price of requiring the use of a log-frequency representation. This causes considerable problems when attempting to resynthesise the separated sources as there is no exact mapping available to map from a log-frequency representation back to a linear-frequency representation, which results in considerable degradation in the sound quality of the separated sources. While refiltering can overcome this problem to some extent, there are still problems with resynthesis.

A further problem with existing techniques was also highlighted, in particular the lack of a strict harmonic constraint on the recovered frequency basis functions. Previous attempts to impose harmonicity used an ad hoc constraint where the basis functions were zeroed in regions where no harmonic activity was expected. While this does guarantee that there will be no activity in these regions, it does not guarantee that the basis functions recovered will have the shape that a sinusoid would have if present in these regions.

Sinusoidal shifted 2D nonnegative tensor factorisation was then proposed as a means of overcoming both of these problems simultaneously. It takes advantage of the fact that a closed form solution exists for calculating the spectrum of a sinusoid of known frequency, and uses an additive-synthesis inspired approach for modeling pitched instruments, where each note played by an instrument is modelled as the sum of a fixed number of weighted sinusoids in harmonic relation to each other. These weights are considered to be invariant to changes in the pitch, and so each note is modelled using the same weights regardless of pitch. The frequency spectrum of the individual harmonics is calculated in the linear frequency domain, eliminating the need to use a log-frequency representation at any point in the algorithm, and harmonicity constraints are imposed explicitly by using a signal dictionary of harmonic sinusoid spectra. Results show that using this signal model results in a better fit to the original mixture spectrogram than algorithms involving the use of a log-frequency representation, thereby demonstrating the benefits of being able to perform the optimisation solely in the linear-frequency domain.

However, it should be noted that the proposed model is not without drawbacks. In particular, best results were obtained if the pitch of the lowest note of each pitched instrument was provided to the algorithm. In most cases this information will not be readily available, and this necessitates the use of the standard shifted 2D nonnegative tensor factorisation algorithm to estimate these pitches before using the sinusoidal model. Research is currently ongoing on other methods to overcome this problem, but despite this, it is felt that the advantages of the new algorithm more than outweigh this drawback.

Using the same harmonic weights or instrument basis function regardless of pitch is only an approximation to the real world situation where the timbre of an instrument does change with pitch. To overcome this limitation, the incorporation of a source-filter model into the tensor factorisation framework had previously been proposed by others. Unfortunately, in the context of sound source separation, it was found that it was difficult to obtain good results using this approach as there were too many parameters to optimise. However, the addition of the strict harmonicity constraint proposed in this paper was found to restrict the range of solutions sufficiently to make the problem tractable.

It had previously been observed that the addition of harmonic constraints was required to create a system which could handle both pitched and percussive instrumentations simultaneously. However, previous attempts at such systems suffered due to the use of log-frequency representations and the lack of a strict harmonic constraint. The combined model presented here extends this earlier work from single channel to multichannel signals, and overcomes these problems by use of sinusoidal constraints applied in the linear-frequency domain, as well as incorporating the source filter model into the system, and so represents a more general model than those previously proposed.

In testing using common source separation performance metrics, the extended algorithms proposed were found to considerably outperform existing tensor factorisation algorithms, with considerably reduced signal distortion and artifacts in the resynthesis. The extended algorithms were also found to be more reliable than SNTF-based approaches.

In conclusion, it has been demonstrated that use of an additive-synthesis based approach for modelling instruments in a factorisation framework overcomes problems associated with previous approaches, as well as allowing extensions to existing models. Future work will concentrate on the improvement of the proposed models, both in terms of increased generality and in improved resynthesis of the separated sources, as well as investigating the effects of the mixing coefficients on the separations obtained. It is also proposed to investigate the use of frequency domain performance metrics as a means of increasing the perceptual relevance of source separation metrics.

## Figures and Tables

**Table 1:** Summary of the tensors used, their dimensions, and function, in the various shift-invariant factorisation models included in this paper. Tensors that occur in multiple models are not repeated.

SNTF	*𝒳*	*r* × *n* × *m*	Signal spectrograms
	$\hat{𝒳}$	*r* × *n* × *m*	Approximation of *𝒳*
	*𝒢*	*r* × *K*	Instrument gains
	*𝒯*	*n* × *z* × *n*	Translation tensor (freq.)
	*𝒜*	*n* × *K* × *p*	Instrument basis functions
	*𝒮*	*z* × *K* × *m*	Note activations
	*𝒫*	*m* × *p* × *m*	Translation tensor (time)

SSNTF	*ℋ*	*n* × *z* × *h*	Harmonic dictionary
	*𝒲*	*h* × *K* × *p*	Harmonic weights

SF-SSNTF	*ℱ*	*n* × *K* × *n*	Formant filters

SF-SSNTF + N	*ℳ*	*r* × *L*	Noise instrument gains
	*ℬ*	*n* × *L* × *q*	Noise basis functions
	*𝒞*	*L* × *m*	Noise activations
	*𝒬*	*m* × *q* × *m*	Noise translation tensor

## References

[1] Stautner JP (1983). *Analysis and synthesis of music using the auditory transform [M.S. thesis]*.

[2] Comon P (1994). Independent component analysis, a new concept?. *Signal Processing*.

[3] Lewicki MS, Sejnowski TJ (2000). Learning overcomplete representations. *Neural Computation*.

[4] Olshausen BA, Field DJ (2004). Sparse coding of sensory inputs. *Current Opinion in Neurobiology*.

[5] Lee D, Seung H (1999). Learning the parts of objects by nonnegative matrix factorisation. *Nature*.

[6] Paatero P, Tapper U (1994). Positive matrix factorization: a non-negative factor model with 
optimal utilization of error estimates of data values. *Environmetrics*.

[7] Casey M, Westner A Separation of mixed audio sources by independent subspace analysis.

[8] Virtanen T Sound source separation using sparse coding with temporal continuity objective.

[9] Smaragdis P, Brown JC Non-negative matrix factorization for polyphonic music transcription.

[10] FitzGerald D, Lawlor B, Coyle E Sub-band independent subspace analysis for drum transcription.

[11] Raczynski S, Ono N, Sagayama S Multipitch analysis with harmonic nonnegative matrix approximation.

[12] Sajda P, Du S, Parra L Recovery of constituent spectra using non-negative matrix factorization.

[13] Virtanen T (2006). *Sound source separation in monaural music signals [Ph.D. thesis]*.

[14] FitzGerald D, Cranitch M, Coyle E Shifted non-negative matrix factorisation for sound source separation.

[15] Mørup M, Hansen LK, Arnfred SM Sparse higher order non-negative matrix factorization.

[16] Abdallah SA, Plumbley MD Polyphonic transcription by non-negative sparse coding of power spectra.

[17] Parry RM, Essa I Incorporating phase information for source separation via spectrogram factorization.

[18] Parry RM, Essa I Phase-aware non-negative spectrogram factorization.

[19] Kompass R A generalized divergence measure for non-negative matrix factorization.

[20] Cichocki A, Zdunek R, Amari S-I Csiszár's divergences for non-negative matrix factorization: family of new algorithms.

[21] Grady PDO (2007). *Sparse separation of under-determined speech mixtures [Ph.D. thesis]*.

[22] FitzGerald D (2004). *Automatic drum transcription and source separation [Ph.D. thesis]*.

[23] Bader BW, Kolda TG (2006). Algorithm 862: MATLAB tensor classes for fast algorithm prototyping. *ACM Transactions on Mathematical Software*.

[24] FitzGerald D, Cranitch M, Coyle E Non-negative tensor factorisation for sound source separation.

[25] Parry RM, Essa I Estimating the spatial position of spectral components in audio.

[26] Barry D, Lawlor B, Coyle E Sound source separation: azimuth discrimination and resynthesis.

[27] Smaragdis P Non-negative matrix factor deconvolution; extraction of multiple sound sources from monophonic inputs.

[28] Virtanen T Separation of sound sources by convolutive sparse coding.

[29] Schmidt MN, Mørup M Nonnegative matrix factor 2-D deconvolution for blind single channel source separation.

[30] Vincent E, Rodet X Music transcription with ISA and HMM.

[31] Nielsen AB, Sigurdsson S, Hansen LK, Arenas-García J On the relevance of spectral features for instrument classification.

[32] Brown JC (1991). Calculation of a constant Q spectral transform. *Journal of the Acoustical Society of America*.

[33] Eggert J, Wersing H, Körner E Transformation-invariant representation and NMF.

[34] FitzGerald D, Cranitch M, Coyle E Shifted 2D non-negative tensor factorisation.

[35] Mørup M, Schmidt MN (2006). Sparse non-negative tensor 2D deconvolution (SNTF2D) for multi channel time-frequency analysis.

[36] FitzGerald D, Cranitch M, Coyle E Sound source separation using shifted non-negative tensor factorisation.

[37] Slaney M (1996). Pattern playback in the 90s. *Advances in Neural Information Processing Systems 7*.

[38] FitzGerald D, Cranitch M, Coyle E Resynthesis methods for sound source separation using non-negative factorisation methods.

[39] FitzGerald D, Cranitch M, Cychowski M Towards an inverse constant Q transform.

[40] Schmidt MN, Mørup M Nonnegative matrix factor 2-D deconvolution for blind single channel source separation.

[41] DeFatta D, Lucas J, Hodgkiss W (1988). *Digital Signal Processing: A System Design Approach*.

[42] Freed A, Rodet X, Depalle P Performance, synthesis and control of additive synthesis on a desktop computer using FFT-1.

[43] Fletcher NF, Rossing TD (1998). *The Physics of Musical Instruments*.

[44] http://csmr.ca.sandia.gov/~tgkolda/TensorToolbox/.

[45] Woodruff J, Pardo B, Dannenberg R Remixing stereo music with score-informed source separation.

[46] Virtanen T, Klapuri A Analysis of polyphonic audio using source-filter model and non-negative matrix factorization.

[47] Välimäki V, Pakarinen J, Erkut C, Karjalainen M (2006). Discrete-time modelling of musical instruments. *Reports on Progress in Physics*.

[48] Schroeder MR, Atal BS Code-excited linear prediction (CELP): high-quality speech at very low bit rates.

[49] Serra X, Poli GD, Picialli A, Pope ST, Roads C (1997). Musical sound modeling with sinusoids plus noise. *Musical Signal Processing*.

[50] Yilmaz Ö, Rickard S (2004). Blind separation of speech mixtures via time-frequency masking. *IEEE Transactions on Signal Processing*.

[51] Siedlaczek P

[52] Vincent E, Gribonval R, Fevotte C (2006). Performance measurement in blind audio source separation. *IEEE Transactions on Audio, Speech and Language Processing*.

[53] http://bassdb.gforge.inria.fr/bss_eval.

